# Role of adaptive radiation therapy for pediatric patients with diffuse pontine glioma

**DOI:** 10.1120/jacmp.v12i2.3421

**Published:** 2011-01-30

**Authors:** Chris Beltran, Saumya Sharma, Thomas E. Merchant

**Affiliations:** ^1^ Department of Radiological Sciences St. Jude Children's Research Hospital Memphis TN USA

**Keywords:** pontine glioma, adaptive radiation therapy

## Abstract

We investigate the role of adaptive radiation therapy in pediatric patients with diffuse pontine glioma and the impact of steroid‐related weight gain on treatment parameters utilizing cone‐beam CT. Fifteen patients with diffuse pontine glioma were treated with three‐dimensional conformal radiation therapy and enrolled on a daily localization protocol. The median age was 6 years (range: 2–13 years). Patient charts were examined to obtain the prescribed daily dose of dexamethasone and weight. The original treatment plan was recalculated based on the data obtained from the daily cone‐beam CT. The changes in target and critical structure doses were calculated using gEUD. Correlations between prescribed dexamethasone, weight gain, source‐to‐skin distance (SSD) changes and dosimetric changes were investigated. Eleven of the 15 patients gained weight during radiation therapy, with an average gain of 2.2 kg (8.0%). The mean gEUD decreased was 0.57 Gy (range: 0.24–1.4 Gy) for the PTV, and the mean gEUD increase for critical structures was 1.14%. No strong correlations between prescribed dexamethasone doses, weight gain and dosimetric changes were found. Change in SSD vs. dose to PTV was correlated $\left(R^{2}=0.51\right)$. Weight gain and changes to the external surface are apparent in these patients; however, the dosimetric changes to the target and critical structures were small and in most cases did not warrant an adaptive plan. The potential exists for a decrease in target dose in these patients; therefore, they should be monitored to assess for replanning when necessary.

PACS number: 87.53Jw

## I. INTRODUCTION

Brainstem tumors account for 10%–15% of all pediatric central nervous system tumors^(^ ^1^ ^–^ ^3^ ^)^ and may be broadly characterized according to their invasiveness and location.^(1)^ The most common and aggressive type is the diffuse intrinsic pontine glioma,^(^ ^1^ ^,^ ^4^ ^)^ for which the prognosis is poor. Median survival times are less than 12 months in most series.^(^ ^1^ ^,^ ^2^ ^,^ ^5^ ^)^

Although not considered curative, radiation therapy is a primary component of treatment. It has the ability to improve symptoms and will extend survival when compared to observation alone.^(6)^ Common symptoms exhibited by these patients include cranial nerve deficits, ataxia and weakness,^(4)^ which may be improved with corticosteroids until the tumor responds to irradiation. The dose and duration of corticosteroid therapy may depend on the extent of disease, severity of symptoms and the responsiveness of the tumor to irradiation.

**Figure 1:**
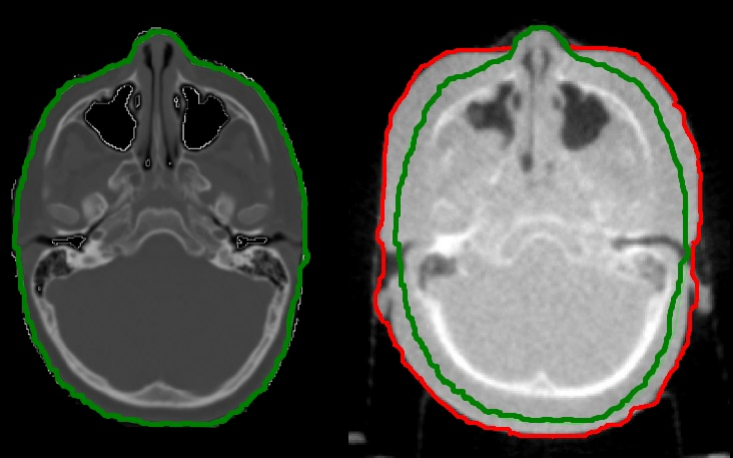
Simulation CT (left) and mid‐therapy CBCT (right). Green (inner) contour is the original body structure and red (outer) is the body structure based on the CBCT. The difference represents the gain in separation due to steroid use and is the structure used in the adaptive calculation.

Although beneficial to the patient, from a practical standpoint corticosteroid therapy may have a negative impact on the administration of radiation therapy. The associated weight gain may affect the shape and volume of the head, as shown in Fig. 1, and thereby impact the distribution of dose. It may be impractical to use the same immobilization device for the entire treatment course, and the localization of the planning target volume may be compromised unless frequent verification is performed to observe for alterations in patient anatomy. Similar concerns have been expressed for adults with head and neck cancer where the external surfaces have shown noticeable change due to tumor shrinkage and patient weight loss.^(^ ^7^ ^–^ ^9^ ^)^

We investigated the impact of steroid‐related weight gain in pediatric patients with diffuse pontine glioma, and evaluated the role of adaptive radiation therapy planning.

## II. MATERIALS AND METHODS

### A. Patient population

Fifteen patients with diffuse pontine glioma were treated with three‐dimensional conformal radiation therapy (CRT) between March 2008 and May 2009, and enrolled on a daily localization protocol^(10)^ that included an investigational low‐dose megavoltage cone‐beam CT (MV‐CBCT) device.^(^ ^11^ ^,^ ^12^ ^)^ This device delivers at most 1 cGy at isocenter per CBCT,^(11)^ which is less than an average port film. The treatment plans consisted of an average of seven fields (range: 6–8). The median age at the time of irradiation was six years (range: 2 to 13 years). Ten patients were treated using IV general anesthesia. The total dose was 54 Gy administered in 30 fractions over a time period of six weeks. All patients were treated in the supine position and immobilized using a thermoplastic face mask. All patients were prescribed dexamethasone at some point during irradiation for the management of symptoms; most often patients required dexamethasone prior to the initiation of treatment and were tapered, when feasible, during the course of treatment. The patient charts were examined to obtain the prescribed daily dose of dexamethasone and patients’ weights. The steroid dose was noted in milligram per day and the weight was recorded in kilograms.

### B. Adaptive planning

For each patient, the original treatment plan was created using the Plan University of North Carolina system (PLUNC) and referred to as the baseline plan. Localization CBCT data from every second treatment day (n=15 per subject) were registered to the original treatment planning CT dataset. Based on the registration, a new structure was created and called “body‐n”, where n represented from which CBCT the registration was taken. The intersection between this body‐n structure and the original body structure was labeled “expansion‐n”. The Hounsfield units within this volume were set to zero, making the new structure water‐equivalent for dose calculation. The treatment plan was then recalculated using the original treatment planning parameters and a new external structure, “external‐n', was created which included ”expansion‐n“. Heterogeneity corrections were used for all plans. The new external contour was used to demonstrate the effect of steroid therapy on the baseline treatment plan and to calculate differences in radiation dose distributions relative to the target and normal tissue volumes. These plans were referred to as modified baseline plans. In addition, an adaptive plan was created in which the target coverage and normal tissue doses were re‐optimized to achieve the goals of the original plan.

### C. Dosimetric analysis

The generalized equivalent uniform dose (gEUD)^(^ ^13^ ^,^ ^14^ ^)^ was used for the dosimetric analysis. The gEUD was calculated for the target as well as the critical structures for the baseline, modified baseline and adaptive plans. The targets of interest were the planning target volume (PTV) and the clinical target volume (CTV); the critical structures were the optic nerves, cochleae, optic chiasm and the spinal cord. A gEUD “a” value of −10 was used for the targets and “a” value of +10 was used for the critical structures except for the cochlea, where an “a” value of +2 was used.^(15)^ In addition, the change in separation was quantified by calculating the average change in source‐to‐surface distance (SSD) between the baseline and modified baseline plans for the treatment ports. Pearson correlation coefficients were calculated. Significance was determined at the *p* ≤ 0.05 level.

## III. RESULTS

**Figure 2:**
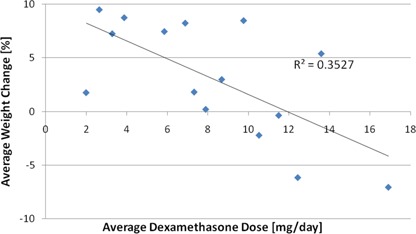
Dexamethasone prescribed dose vs. weight change.

**Figure 3:**
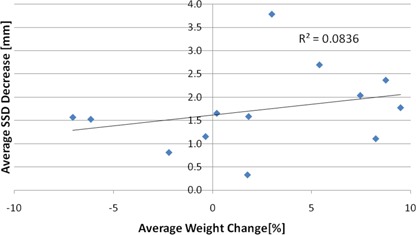
Average weight change compared to the decrease in SSD.

**Figure 4:**
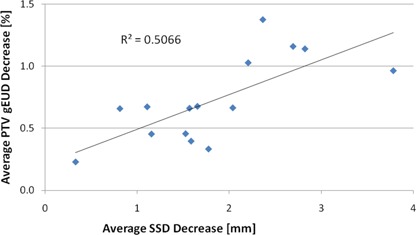
The average decrease in SSD compared to the average percent decrease in the gEUD of the PTV for each patient.

**Figure 5:**
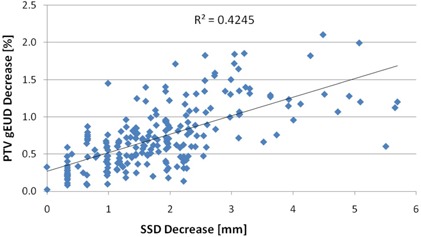
The decrease in SSD for a single treatment compared to the percent gEUD decrease of the PTV for that treatment.

In this study, the average patient weighed 30.2 kg at the start of irradiation and gained 2.2 kg (8.0%) by the completion of the six‐week treatment course. Weight gain was inversely related to steroid dose (Fig. 2); however, this relationship was not statistically significant $\left(R^{2}=0.35\right)$. The average decrease in SSD for all fields based on each CBCT examined was 1.8 mm (range: 0.0–5.7 mm). The average maximum SSD change for the 15 patients was 2.9 mm (range: 0.3–5.7 mm). There was no correlation between the change in SSD and weight $(R^{2}=0.08;$ Fig. 3); however, as shown in Fig. 4, there was a correlation between average change in SSD and gEUD of the PTV for the modified baseline plans $(R^{2}=0.51,p=0.02$). Figure 5 shows the correlation between SSD and gEUD change for each of the 225 modified baseline plan $\left(R^{2}=0.42,p<0.001\right)$. Seven of the 15 patients had shims placed under the mask to accommodate increasing head size and two patients needed to have a part of the mask cut away to use their immobilization device. In this cohort, no patients required a new thermoplastic mask.

The mean gEUD decrease of the modified baseline plans was 0.39 Gy (range 0.12 to 0.74 Gy) for the PTV, the mean gEUD change for all critical structures was 1.14%. Table 1 lists the percent decrease in gEUD dose from the baseline plan to modified baseline plan for all the structures of interest. Individual patient analysis showed decrease in the gEUD of the PTV for every patient with a maximum decrease of 1.4 Gy. The difference in gEUD between the baseline and adaptive plans for all structures was less than 0.5%.

**Table 1:** Planned and delivered gEUD for target and normal structures.

	*Average Baseline*	Average Modified	*Percent Change*
*Structure*	gEUD (Gy)	Baseline gEUD (Gy)	*(%)*
PTV	54.29	53.72	−1.04
CTV	54.33	53.78	−1.01
Left Optic Nerve	17.68	17.49	0.94
Right Optic Nerve	17.25	16.95	1.44
Optic Chiasm	47.69	47.19	1.04
Spinal Cord	48.25	47.62	1.31
Left Cochlea	49.20	48.66	1.07
Right Cochlea	46.64	46.15	1.02

## IV. DISCUSSION

We did not find any significant relationship between daily corticosteroid dose and weight gain during the six weeks of radiation therapy. The lack of correlation may reflect the lasting impact of steroid therapy despite efforts to taper the medication during treatment. All patients showed a decrease in SSD values, including those that lost body weight, reflecting the cushingoid effect of steroids on the face and head. The dosimetric analysis showed that, on average, the dose to the target over the entire course of treatment was decreased by 1%, and at most by 1.4%. Although this change was statistically significant (p<0.001), clinically this will likely not have an impact on the treatment. A reasonable threshold for creating an adaptive plan is a loss of coverage of 1.5% for the entire treatment or 2% for an individual fraction. No patent had a decrease of more than 1.4% for the entire treatment and only one patient had individual treatments where the target gEUD decreased more than 2% (2.0% and 2.1%). In this unique case, these treatments were during the last week of therapy and did not warrant an adaptive plan. In general, this type of steroid induced cushingoid effect would have the largest impact on brain tumors located in the posterior fossa region, such as the pontine gliomas studied here. This is because the SSD changes tend to be prominent in the face and base of skull region; therefore, targets in other areas of the brain should be impacted less than described here. Although no patient required a new thermoplastic mask in this study, we have had instances where a new mask was required.

## V. CONCLUSIONS

Weight gain and changes to the external surface are apparent in patients with diffuse intrinsic pontine glioma; however, the dosimetric changes to the target and critical structures are small and rarely warrant an adaptive plan. The potential exists for a decrease in the target dose; therefore, these patients should continue to be monitored and assessed for replanning when necessary.

## ACKNOWLEDGMENTS

This work was supported in part by the Pediatric Oncology Education Program (NCI grant #5 R25 CA23944), Siemens Medical Systems, and the American Lebanese Syrian Associated Charities (ALSAC).

## References

[1] Freeman CR , Farmer JP . Pediatric brain stem gliomas: a review. Int J Radiat Oncol Biol Phys. 1998;40(2):265–71. 9457808 10.1016/s0360-3016(97)00572-5

[2] Frazier JL , Lee J , Thomale UW , Noggle JC , Cohen KJ Jallo GI . Treatment of diffuse intrinsic brainstem gliomas: failed approaches and future strategies. J Neurosurg Pediatr. 2009;3(4):259–69. 19338403 10.3171/2008.11.PEDS08281

[3] Fangusaro J . Pediatric high‐grade gliomas and diffuse intrinsic pontine gliomas. J Child Neurol. 2009;24(11):1409–17. 19638636 10.1177/0883073809338960

[4] Korones DN . Treatment of newly diagnosed diffuse brain stem gliomas in children: in search of the holy grail. Expert Rev Anticancer Ther. 2007;7(5):663–74. 17492930 10.1586/14737140.7.5.663

[5] Lewis J , Lucraft H Gholkar A . UKCCSG study of accelerated radiotherapy for pediatric brain stem gliomas. United Kingdom Childhood Cancer Study Group. Int J Radiat Oncol Biol Phys. 1997;38(5):925–29. 9276356 10.1016/s0360-3016(97)00134-x

[6] Korones DN , Fisher PG , Kretschmar C , et al. Treatment of children with diffuse intrinsic brain stem glioma with radiotherapy, vincristine and oral VP‐16: a Children's Oncology Group phase II study. Pediatr Blood Cancer. 2008;50(2):227–30. 17278121 10.1002/pbc.21154

[7] Foroudi F , Wong J , Haworth A , et al. Offline adaptive radiotherapy for bladder cancer using cone beam computed tomography. J Med Imaging Radiat Oncol. 2009;53(2):226–33. 19527372 10.1111/j.1754-9485.2009.02066.x

[8] Paquin D , Levy D Xing L . Multiscale registration of planning CT and daily cone beam CT images for adaptive radiation therapy. Med Phys. 2009;36(1):4–11. 19235367 10.1118/1.3026602 PMC2739311

[9] Ding GX , Duggan DM , Coffey CW , et al. A study on adaptive IMRT treatment planning using kV cone‐beam CT. Radiother Oncol. 2007;85(1):116–25. 17707938 10.1016/j.radonc.2007.06.015

[10] Beltran C , Krasin MJ Merchant TE . Inter‐ and intrafractional positional uncertainties in pediatric radiotherapy patients with brain and head and neck tumors. Int J Radiat Oncol Biol Phys. 2010;In Press. 10.1016/j.ijrobp.2009.12.057 PMC3536549 20605345

[11] Beltran C , Lukose R , Gangadharan B , Bani‐Hashemi A , Faddegon BA . Image quality & dosimetric property of an investigational imaging beam line MV‐CBCT. J Appl Clin Med Phys. 2009;10(3):3023. 10.1120/jacmp.v10i3.3023 PMC5720554 19692984

[12] Faddegon B , Wu V , Pouliot J , Gangadharan B , Bani‐Hashemi A . Low dose megavoltage cone beam computed tomography with an unflattened 4 MV beam from a carbon target. Med Phys. 2008;35(12):5777–86. 19175135 10.1118/1.3013571

[13] Niemierko A . Reporting and analyzing dose distributions: a concept of equivalent uniform dose. Med Phys. 1997;24(1):103–10. 9029544 10.1118/1.598063

[14] Niemierko A . A generalized concept of equivalent uniform dose (EUD) [abstract]. Med Phys. 1999;26:1100.

[15] Wu Q , Mohan R , Niemierko A , Schmidt‐Ullrich R . Optimization of intensity‐modulated radiotherapy plans based on the equivalent uniform dose. Int J Radiat Oncol Biol Phys. 2002;52(1):224–35. 11777642 10.1016/s0360-3016(01)02585-8

